# Comparative transcriptome analysis of *Lupinus polyphyllus* Lindl. provides a rich molecular resource for research on coloration mechanism

**DOI:** 10.7717/peerj.13836

**Published:** 2022-08-02

**Authors:** Zhu Gao, Jipeng Mao, Lu Chen, Xiaoling Wang, Lu Zhang

**Affiliations:** 1 College of Forestry, Jiangxi Agricultural University, Nanchang, Jiangxi, China; 2 Institute of Biological Resources, jiangxi Academy of Sciences, Nanchang, Jiangxi, China; 3 Jinggangshan Institute of Biotechnology, Nanchang, Jiangxi, China

**Keywords:** *Lupinus polyphyllus*, Transcriptome, Anthocyanin, Carotenoid, Genes

## Abstract

*Lupinus polyphyllus* is rich in color, making it a well-known horticultural ornamental plant. However, little is known about the genes related to anthocyanin and carotenoid biosynthesis in *L. polyphyllus*. In this study, transcriptome sequencing was performed on eight different colors of* L. polyphyllus*. A total of 1.13 billion clean reads were obtained and assembled into 89,124 unigenes, which were then aligned with six databases, resulting in the identification of 54,823 annotated unigenes. Among these unigenes, 76 and 101 were involved in the biosynthetic pathway of carotenoids and anthocyanins, respectively. In addition, 505 transcription factors were revealed, which belonged to the *MYB*, *R2R3-MYB*, *NAC*, *bHLH*, and *WD40* families. A total of 6,700 differentially expressed genes (DEGs) were obtained by comparative transcriptome analysis. Among them, 17 candidate unigenes (four carotenoid genes, seven anthocyanin genes, and six TFs) were specifically up-regulated for one or more colors of *L. polyphyllus*. Eight representative candidate unigenes were analyzed by qRT-PCR. The findings enrich the transcriptome database of lupine, and provide a rich molecular resource for research on the coloration mechanism of *L. polyphyllus*.

## Introduction

Lupine (*Lupinus sp*.), a member of the genistoid clade of the Fabaceae family, comprises an uncertain number of annual or perennial plants and more than 500 species worldwide (Bermudez-Torres et al., 2009). It has great ornamental value owing to its rich color and long flowering period. Lupine can also play important roles in crop rotation and organic farming, as it can fix free nitrogen in the atmosphere and effectively absorb phosphorus from the soil via diazotrophic symbiosis and formation of cluster roots (Lambers, Clements & Nelson, 2013; Watt & Evans, 2003; Schulze et al., 2006). Also, lupine is a rich source of energy, fiber, oil, micronutrients, and bioactive non-nutrient compounds, and its seeds are a valuable source of protein as well (Burgos-Díaz et al., 2019). Some studies have demonstrated various nutraceutical properties of lupine, such as reduction of hyperglycemia, hypercholesterolemia, and hypertension (Arnoldi & Greco, 2011). Therefore, lupine is a promising alternative for soy proteins in food products due to its high protein content and potential health benefits. However, lupine is not as popular as other legumes, possibly due to its high contents of bitter and potentially toxic quinolizidine and piperidine alkaloids in seeds (Green et al., 2013). This problem has been solved in many modern lupine cultivars (Kroc et al., 2017). *Lupinus polyphyllus* is a perennial plant with a variety of colors including red, white, yellow, purple, pink and tertiary colors. It was introduced into China in the 1990s as a horticultural ornamental plant. Currently, most studies of *L. polyphyllus* have been focused on its effects on the ecological environment (Hensgen & Wachendorf, 2016; Hassani et al., 2021). However, there are few studies of the color and anthocyanidins composition of *L. polyphyllus*. Only Dp3-malonylglucosid and Cy3-malonylglucosid have been identified in the blue and pink flowers of *L. polyphyllus* (Takedal, Jeffrey & Peter, 1993).

Flower color is an important trait and target for the breeding of ornamental plants, which is closely associated with the accumulation of anthocyanins, carotenoids, and betalains (Grotewold, 2006). Anthocyanins confer the colors of orange, pink, red, purple, and blue; carotenoids contribute to the colors of yellow to red; and betalains confer the colors of yellow, orange, red, and purple in Caryophyllales plants (Tanaka, Sasaki & Ohmiya, 2008; Jiao et al., 2020). The biosynthetic pathway of anthocyanins has been well characterized in *Arabidopsis thaliana* (Qi et al., 2011), *Medicago truncatula* (Pang et al., 2007), and *Zea mays* (Morohashi et al., 2012), and is a part of flavonoid branch within the phenylpropanoid pathway. The first step in anthocyanin biosynthesis is the formation of 4,2′, 4′,6-tetrahydroxychalcone from 4-coumaroyl-Coa by chalcone synthase (CHS), which is subsequently transformed into naringenin under the catalysis of chalcone isomerase (CHI). The naringenin is then converted to eriodictyol and dihydrokaempferol under the catalysis of flavonoid 3′-hydroxylase (F3′H) and flavanone 3-hydroxylase (F3H), respectively. In addition, anthocyanidin 5-O-glucoside-6-O-malonyltransferase (*5MaT1*), leucoanthocyanidin reductase (*LAR*), anthocyanidin 3-O-glucosyltransferase (*BZ1*), anthocyanidin 3-O-glucoside 2-O-glucosyltransferase (*GT*), dihydroflavonol reductase (*DFR*), anthocyanidin synthase (*ANS*), anthocyanidin reductase (*ANR*), and UDP-glucose flavonoid 3-o-glucosyltransferase (*UFGT*) are also involved in the biosynthesis of anthocyanin (Xie et al., 2003; Davies & Schwinn, 2010; Jaakola, 2013). The biosynthesis of anthocyanins is regulated by transcription factors (TFs) from several different families, including *R2R3-MYB*, *bHLH*, *WD40* and *NAC* (Ramsay & Glover, 2005; Allan, Hellens & Laing, 2008; Zhou et al., 2015; Dasgupta et al., 2017). The MBW protein complexes containing MYB, bHLH, and WD40 repeat factors are important transcriptional regulators of anthocyanins, and mainly involved in the regulation of late flavonoid biosynthesis genes (Gao et al., 2020; Xu et al., 2021). Previous studies have revealed that the expression abundance of anthocyanin biosynthesis-related structural genes is directly regulated by the conserved MBW complex. In addition, the MBW complex is involved in proanthocyanin biosynthesis, trichome formation, vacuolar acidification, seed coat differentiation and other developmental events (Li et al., 2020a; Li et al., 2020b; Xu, Dubos & Lepiniec, 2015).

The biosynthetic pathway of carotenoids is initiated through condensation of geranylgeranyl pyrophosphate to phytoene under the catalysis of phytoene synthase (PSY) (Kato et al., 2004). Phytoene is then converted to lycopene by phytoene desaturase (PDS), 15-cis-zeta-carotene isomerase (Z-ISO), zeta-carotene desaturase (ZDS), and carotenoid isomerase (CRTISO). From lycopene, the pathway is further bifurcated into two crucial branches. Lycopene *ɛ*-cyclase (LCYE) is responsible for the synthesis of *δ*-carotene and *α*-carotene (*α*-branch), and lycopene *β*-cyclase (LCYB) is responsible for that of *γ*-carotene and *β*-carotene (*β*-branch) from lycopene, respectively. The *α*-carotene is then converted into lutein under the catalysis of *ɛ*-ring hydroxylase and *β*-ring hydroxylase, and *β*-carotene is transformed into zeaxanthin under the catalysis of *β*-carotene hydroxylase (BCH). Subsequently, neoxanthin is formed from zeaxanthin under the catalysis of zeaxanthin epoxidase (ZEP) and neoxanthin synthetase (NXS). Finally, the end product abscisic acid is generated from violaxanthin through a reaction mediated by 9-cis-epoxycarotenoid dioxygenase (NCED) (Cunningham et al., 1996; Kato et al., 2004; Karanjalker et al., 2017; Wang et al., 2020). In addition, several members of the *R2R3-MYB* and *bHLH* TF families have been reported to regulate carotenoid biosynthesis (Zhou, Jiang & Yu, 2011; Endo, Fujii & Sugiyama, 2016; Zhu et al., 2017; Ampomah-Dwamena, Thrimawithana & Dejnoprat, 2019).

Transcriptome sequencing (RNA-Seq) technology can greatly facilitate the discovery and identification of genes involved in metabolite biosynthesis, particularly for those species without reference genomes. Many genes involved in the biosynthesis of anthocyanins and carotenoids in plants have been successfully discovered by RNA-Seq (Mao et al., 2021), such as anthocyanin-related genes in *Camellia reticulata* (Yao et al., 2016), *Vitis davidii* (Sun et al., 2016), *Rosa rugosa* (Li et al., 2018), *Curcuma longa* (Sahooa et al., 2019), *Raphanus sativus* (Liu & Chen, 2019), and strawberry (Lin et al., 2021), and carotenoid-related genes in *Momordical cochinchinensis* (Hyun et al., 2012), *Lycium chinense* (Wang et al., 2015), *Mangiferaindica* (Karanjalker et al., 2017), *Citrus maxima* (Wang et al., 2020), and *Brassica napus* (Jia et al., 2021). In this study, transcriptome sequencing and comparative analysis were performed on *L. polyphyllus* with eight colors, which are the most widely used in ornamental horticulture, aiming to dissect the mechanism for the coloration of this special plant. The results might enrich the plant omics database and provide a resource for creating germplasms of *L. polyphyllus* with new flower colors.

## Material and Methods

### Plant materials and RNA isolation

**Figure 1:**
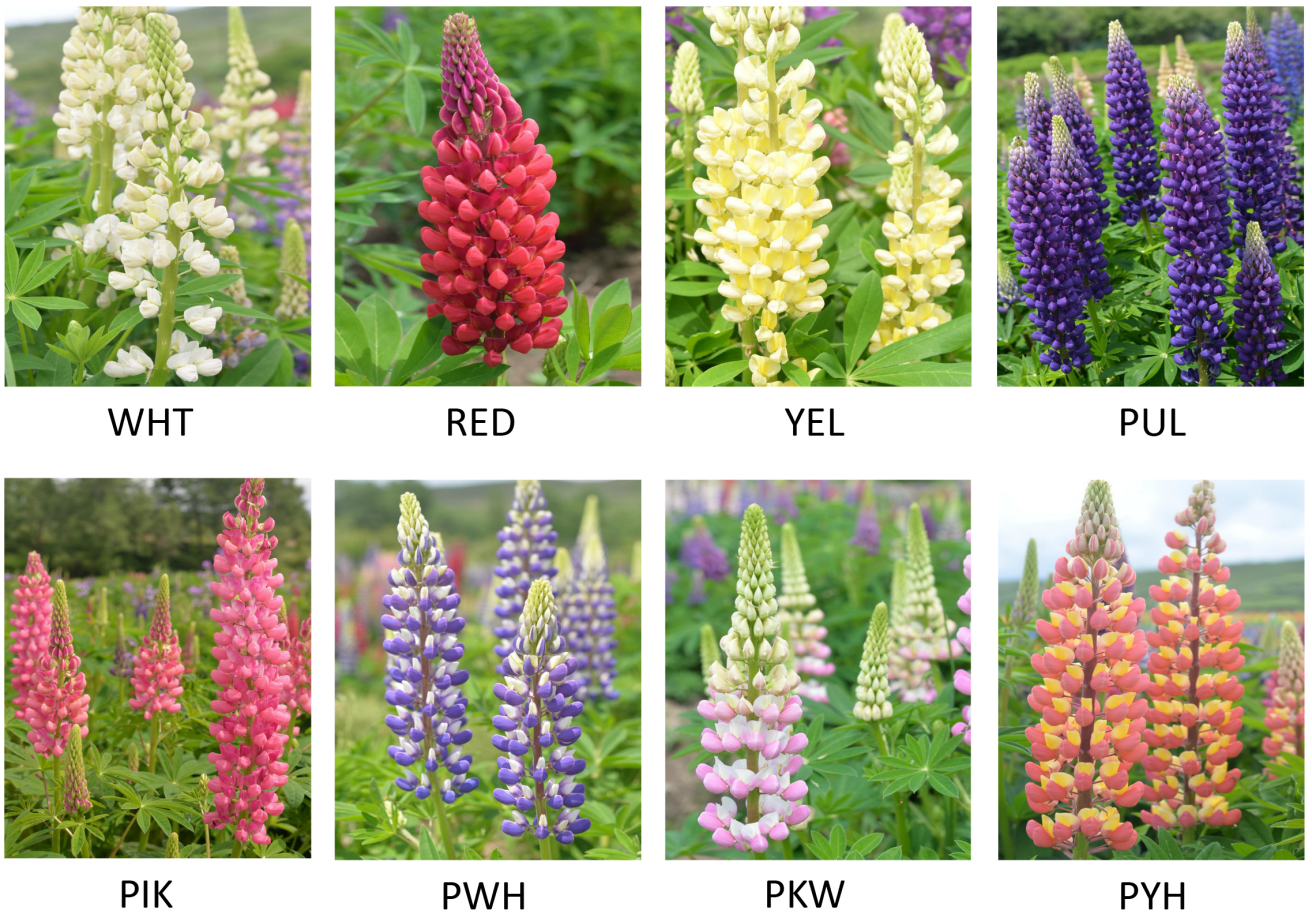
Eight colors of *L. polyphyllus* for study. The WHT, RED, YEL, PUL, PIK, PWH, PKW, and PYH represented the white, red, yellow, purple, pink, purple-white, pink-white, and pink-yellow flowers, respectively.

One-year-old *L. polyphyllus* plants in Fengxin County Doctor kiwifruit Base (E114°45′, N28°34′), Yichun City, Jiangxi Province, China with same environmental conditions and free of pests and diseases were selected for the study. The petals in full bloom of *L. polyphyllus* with eight different colors (white (WHT), red (RED), yellow (YEL), purple (PUL), pink (PIK), purple-white (PWH), pink-white (PKW), and pink-yellow (PYH)), which had consistent flowering period, were collected separately at the same time during flowering period (April) (Fig. 1). Three biological replicates were collected for each color, with each repeat consisting of nine *L. polyphyllus* petals. The fresh petals were immediately frozen in liquid nitrogen and stored at −80 °C until total RNA extraction and subsequent analysis. Total RNA was extracted from each sample with an EASY Spin Plant RNA Extraction Kit (Aidlab Biotechnologies Co., Ltd., Gdansk, Pomorskie, Poland) according to the manufacturer’s instructions. The concentration and quality of each RNA sample were determined by NanoDrop 2000™ micro-volume spectrophotometer (Thermo Scientific,Waltham, MA, USA).

### Illumina sequencing and de novo assembly

The cDNA libraries were constructed following the protocol described by Foucart et al. (2006). RNA-Seq analysis was performed by the Shanghai Majorbio Bio-pharm Technology Co., Ltd. (Shanghai, China) based on Illumina Novaseq6000 platform. The Fastp v0.23.2 software was used to assess the quality of the raw reads (Chen et al., 2018). After filtering of the reads containing adapter or ploy-N and low quality reads from the raw data, the software of Trinity (Haas et al., 2013) was used to assemble high-quality reads into unigenes. At first, assembly based on *Lupinus angustifolius* (available at NCBI ID: 11024) (Yang et al., 2013) was considered. However, the assembly was not successful due to the poor quality of its genome (191 thousands contigs).

### Functional annotation and classification

Unigene sequences were queried against the Non-redundant (Nr), Swiss-Prot and Clusters of Orthologous Groups of proteins (COG) databases with an *E*-value threshold <10 ^−5^, and against the Kyoto Encyclopedia of Genes and Genomes (KEGG), Pfam, and Gene Ontology (GO) databases with default parameters to obtain the functional annotations. The highest sequence similarity to a given unigene was defined as its protein functional annotation. The GO functional classification and KEGG pathway analysis were conducted using the Web Gene Ontology Annotation Plot (WEGO) (Ye et al., 2006) and KEGG automatic annotation server, respectively.

### Differential expression analysis and candidate gene identification

The expression levels of the unigenes were normalized and calculated as the value of fragments per kilobase of transcripts per million mapped fragments (FPKM). The differentially expressed genes (DEGs) were identified as previously described by Anders & Huber (2010). Specifically, a threshold of *p* < 0.05 and a greater-than-fourfold change (absolute value of log2 ratio ≥ 2) were set. The KEGG automatic annotation was performed to identify the genes related to anthocyanin and carotenoid biosynthetic pathways. The genes involved in regulating anthocyanin and carotenoid biosynthesis were revealed based on functional annotation and literature. DEG clustering was also performed to select the candidate genes (Sun et al., 2016).

### Quantitative real-time PCR analysis

A total of eight candidate genes were selected to investigate the expression profiles by qRT-PCR analysis. The primers for the selected candidate genes were designed by Primer Premier 5.0 software, and the *LpActin* gene (GeneBank accession number: XM_019593088.1) was used as the internal reference to correct the expression levels of candidated genes. The qRT-PCR analysis was performed using the LightCycler 480 System (Roche, Basel, Switzerland) and a TB Green Premix Ex Taq II kit (Code No: RR820A, TaKara). The amplification conditions were set according to the description of Wang et al. (2014): one cycle of 95 °C for 30 s, followed by 40 cycles of 95 °C for 5 s, 55 °C for 30 s, and 72 °C for 30 s. Three technical replicates were performed for each qRT-PCR reaction. The relative expression levels of the candidate genes were calculated with the previously described method of 2^−ΔΔ*CT*^in Livak & Schmittgen (2001).

## Results

### RNA-Seq, de novo assembly and functional annotation

**Figure 2:**
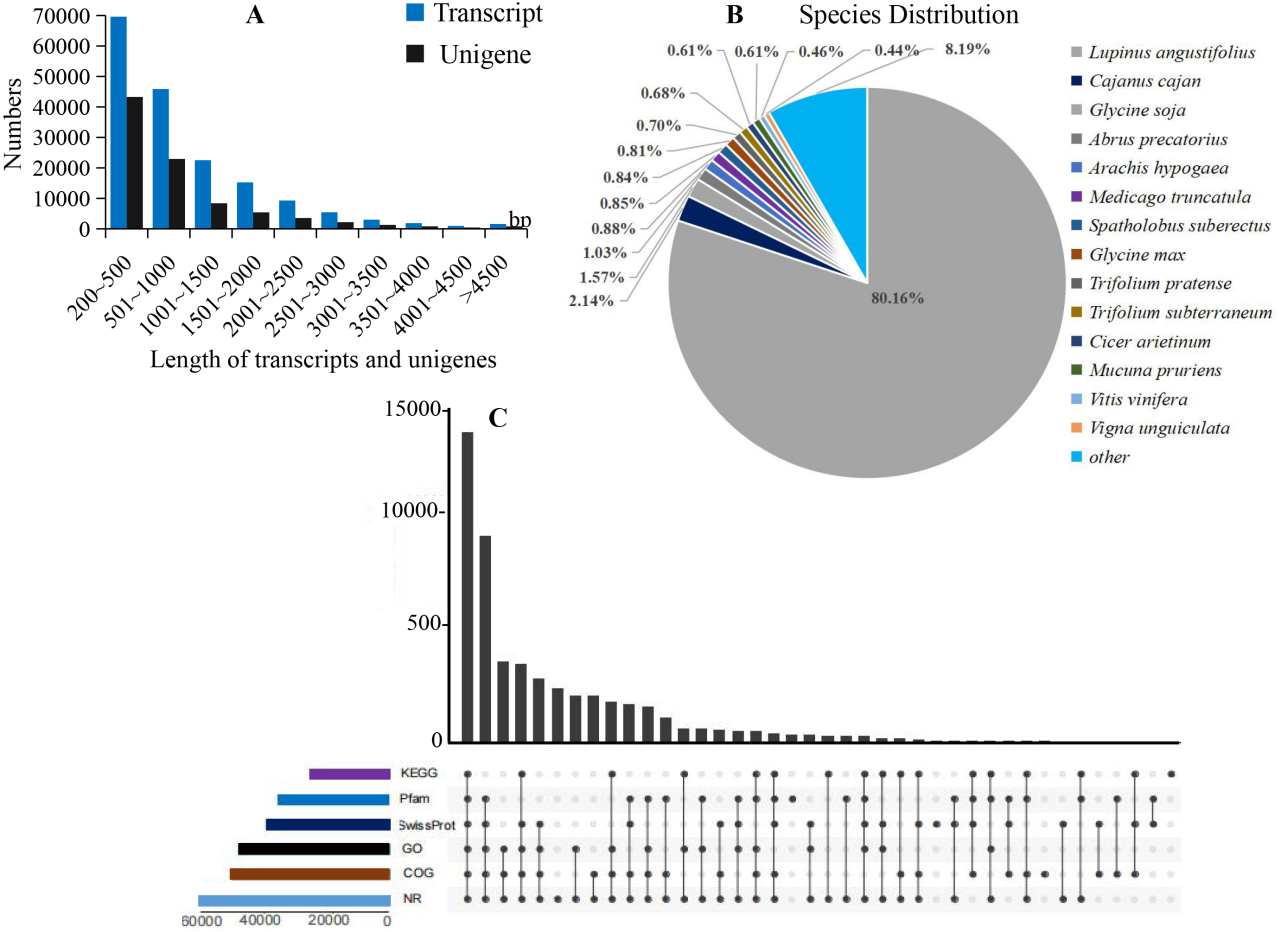
Summary of the assembly and annotations. (A) Sequence length distribution of unigenes and transcripts obtained from eight colors of *L. polyphyllus*. (B) Species distribution of unigenes annotated in Nr database. (C) Annotation information obtained from six different databases.

In total, 24 cDNA libraries of petals from WHT, RED, YEL, PIK, PUL, PKW, PWH, and PYH colors of *L. polyphyllus* were constructed and sequenced on the Novaseq6000 platform (Table S1). A total of 1,152,209,716 raw reads were obtained, with an average Q30 of 93.86% and a mean GC content of 43.13%. The raw reads of the libraries have been deposited in the NCBI Sequence Read Archive (SRA) database (accession number: PRJNA783465). After filtering of the low quality reads, adaptor sequence fragments and empty reads from raw data, a total of 1,132,877,570 clean reads were generated with an average Q30 of 94.45% and a mean GC content of 42.82%. A total of 175,392 transcripts and 89,124 unigenes (Table S2) were assembled from the clean reads based on the Trinity platform. The average length of the transcripts and unigenes was 998 bp and 868 bp, and the GC content was 38.76% and 38.24%, respectively (Fig. 2A). All the assembled unigenes were annotated against six protein databases via a Basic Local Alignment Search Tool (BLASTx) with *E*-value ≤ 10^−5^.

**Figure 3:**
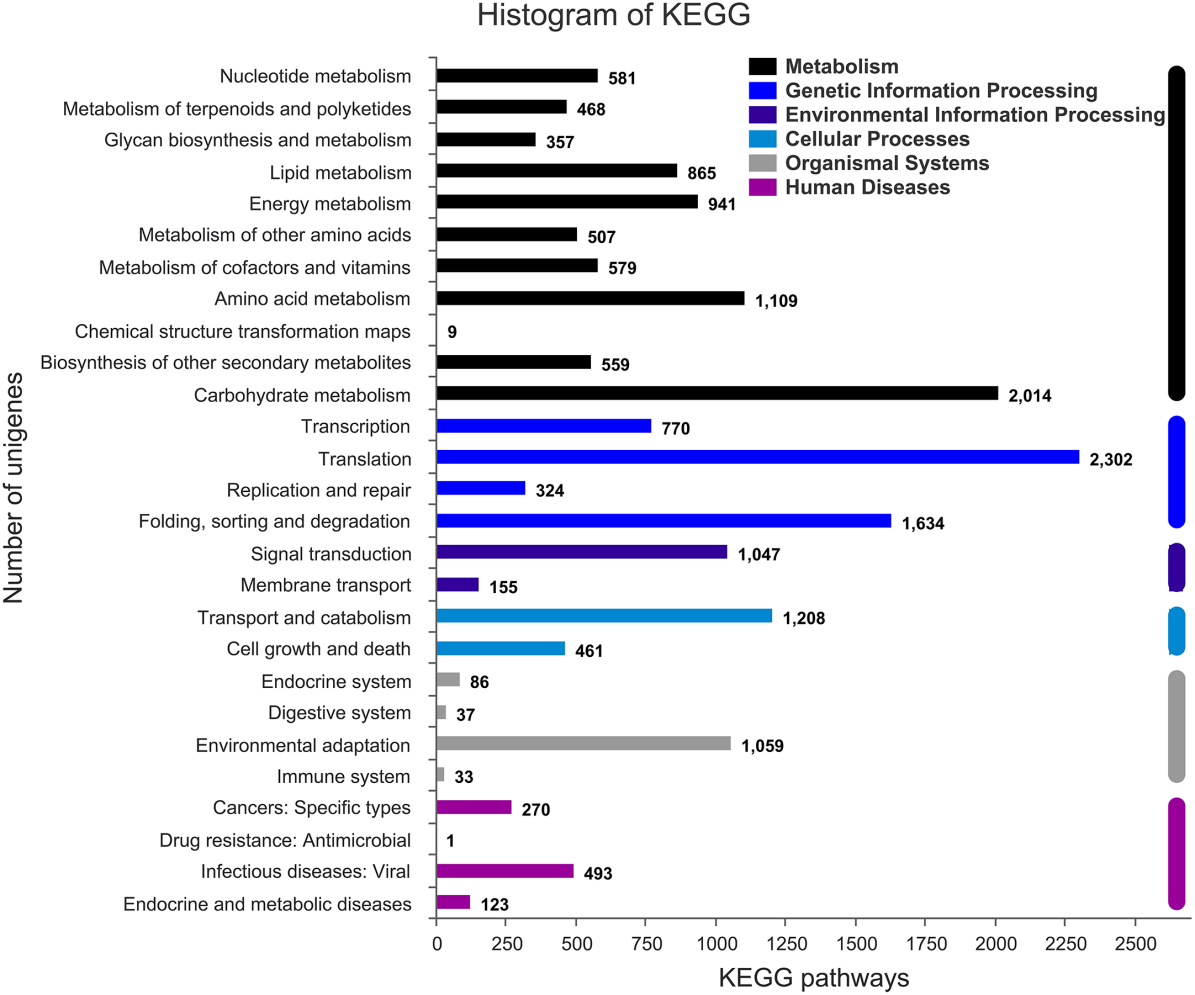
Assembled unigenes were functionally classificated by KEGG classification. The unigenes corresponded to six main categories: Cellular Processes, Environmental Information Processing, Genetic Information Processing, Metabolism, Organismal Systems, and Human Diseases.

The annotation information of a total of 54,823 (61.63%) unigenes was obtained (Table S2). In summary, 53,974 (60.67%), 45,863 (51.55%), 43,972 (49.43%), 35,942 (40.40%), 32,603 (36.65%), and 23,487 (26.40%) unigenes were annotated in the Nr, COG, GO, Swiss-Prot, Pfam and KEGG databases, respectively (Fig. 2C). Based on the Nr database, 43,303 (80.16%) unigenes exhibited higher homology with sequences from *L. angustifolius* (Fig. 2B). The unigenes annotated in the GO database were mainly distributed in 52 terms of three categories. In the biological process, the dominant GO terms included ‘cellular process’ and ‘metabolic process’. Within the category of cellular component, the most enriched GO terms were ‘cell part’, ‘membrane part’ and ‘organelle’. The molecular function category comprised ‘binding’ and ‘catalytic activity’ as the dominant GO terms (Fig. S1). The unigenes matched with the KEGG database were mainly assigned to 146 pathways of 27 sub-categories. The pathway with the largest number of annotated unigenes was the ‘ribosome’ pathway (937 unigenes), followed by the ‘RNA transport’ (778 unigenes) and ‘endocytosis’ pathways (706 unigenes) (Fig. 3 and Table S3).

### DEG identification and analysis

**Figure 4:**
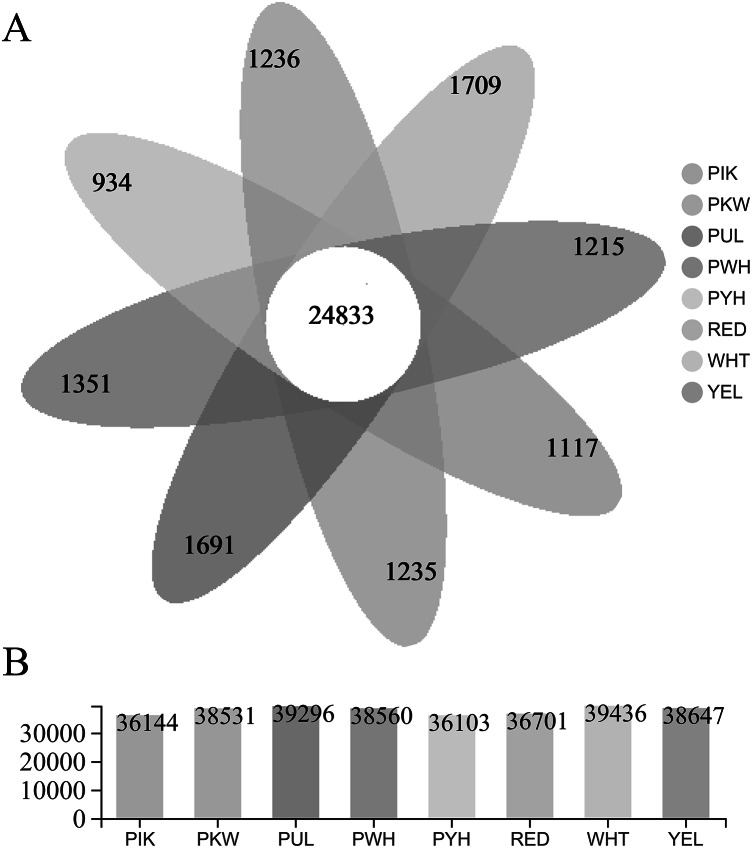
Statistical analysis of unigenes. (A) Number of expressed unigenes for eight colors of *L. polyphyllus*. (B) The Venn analysis, the number in different colors of Venn indicate the unigenes of different *L. polyphyllus* petal tissue-specific expression.

**Figure 5:**
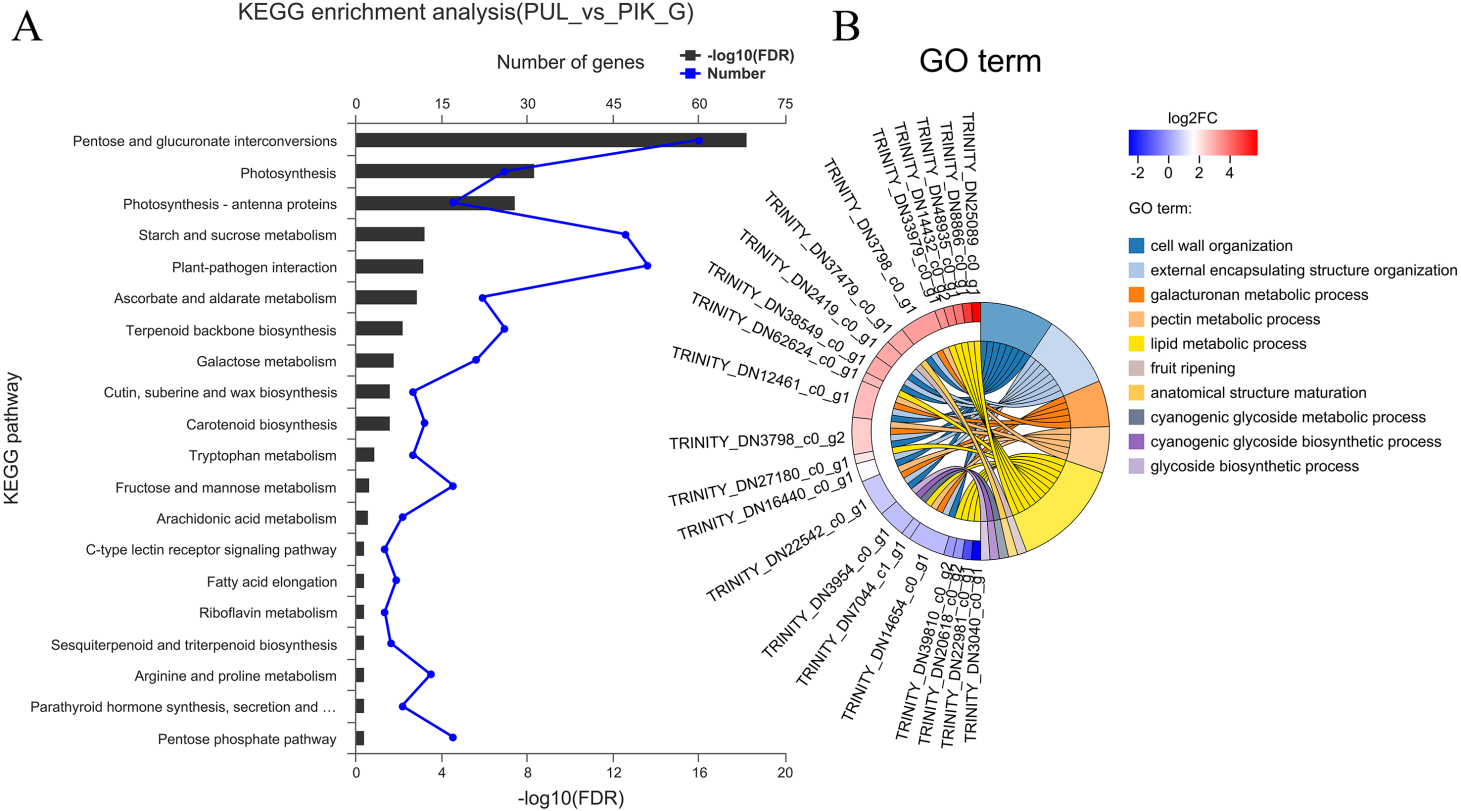
Enrichment analysis of differentially expressed genes obtained in the PUL_vs_PIK comparison group. (A) KEGG enrichment analysis, only KEGG pathway with the top 20 enriched genes were displayed. (B) GO term analysis, only GO terms with top 10 enriched genes were displayed.

The FPKM value was calculated for each unigene, and significant DEGs were screened by setting —log2 ^(foldchange)^— ≥ 2 and *P* ≤ 0.05 as the thresholds. The average number of expressed unigenes in the eight color groups was 37,927. The WHT color group had the largest number of expressed unigenes (39,436), among which 1,709 unigenes were specifically expressed in this group. The PYH color group had the smallest number of expressed unigenes (36,103), among which 934 were expressed (Fig. 4). In addition, a total of 6,700 significant DEGs were obtained from 28 comparison groups of the eight colors (Table S4). The largest number of significant DEGs were observed in the PIK_vs_PUL comparison group (1,721 up-regulated; 1,029 down-regulated), followed by the PIK_vs_RED, PUL_vs_WHT and PYH_vs_PUL comparison groups. However, only 171, 161 and 142 significant DEGs were detected in YEL_vs_PYH, PKW_vs_PYH, and YEL_vs_PKW comparison groups, respectively (Table S5). To better understand the biological functions of the DEGs, the representative significant DEGs obtained in PIK_vs_PUL were analyzed by GO and KEGG analysis. For GO analysis, the significant DEGs were mainly enriched in ‘lipid metabolic process (GO:0006629)’, ‘external encapsulating structure organization (GO:0045229)’, and ‘cell wall organization (GO:0071555)’ (Fig. 5B). The KEGG pathway enrichment analysis showed that these significant DEGs were mainly involved in the ‘pentose and glucuronate interconversions (map00040)’, ‘plant-pathogen interaction (map04626)’, and ‘plant hormone signal transduction (map04075)’ pathways (Fig. 5A).

### Identification and analysis of unigenes related to anthocyanin and carotenoid biosynthesis

Based on the functional annotation and KEGG pathway analysis, a total of 76 and 101 unigenes were revealed to be associated with carotenoid and anthocyanin biosynthesis, respectively (Table S6). Among them, six unigenes (*PSY2*, *NCED1*, *BCH1*, *BCH2*, *ZEP*, and *ZDS*) related to carotenoid biosynthesis and 23 unigenes (*LDOX1*, *LDOX2*, 2 *5MaT1*, *GT*, 3 *ANR*, *BZ1.2*, *BZ1.7*, *3MaT1*, *LAR*, 2 *CHS*, *CHS1A*, *FLS*, 3 *DRF*, *CHI*, *F3′H*, *F3′5′H*, and *C4H*) related to anthocyanin biosynthesis showed differential expression in one or more comparison groups (Table S7). In addition, a total of 505 unigenes were annotated as TFs, which have been reported to be involved in regulating carotenoid and/or anthocyanin biosynthesis, including 19 *WD40*, 19 *R2R3-MYB*, 88 *NAC*, 122 *bHLH*, and 256 *MYB* TFs. Among these 505 TFs, 79 showed differential expression in one or more comparison groups (Table S7). Therefore, a total of 108 DEGs (six related to carotenoid biosynthesis, 23 related to anthocyanin biosynthesis, and 79 TFs) were speculated to be associated with the color of *L. polyphyllus*. K-means analysis of these 108 DEGs revealed that a total of 17 unigenes (four carotenoid genes, seven anthocyanin genes, and six TFs) were specifically up-regulated for one or more colors.

Among them, the specifically up-regulated unigenes included *BCH1*, *BCH2* and *ZEP* in PIK and PYH, *NCED1* in PKW, PUL and PWH, *ANS* and *GT* in PIK, PKW and WHT, *ANR* and *CHS1A* in PYH and PIK, *CHS* and *DFR* in PIK and WHT, *F3′H* in PUL, WHT and YEL. The specifically up-regulated TFs are *NAC2* and *MYB3* in WHT, *MYB48* in PUL, *WD40* in PIK and WHT, *MYB113* in PIK and PYH, and *NAC50* in RED and WHT.

### Quantitative RT-PCR validation of differential gene expression

**Figure 6:**
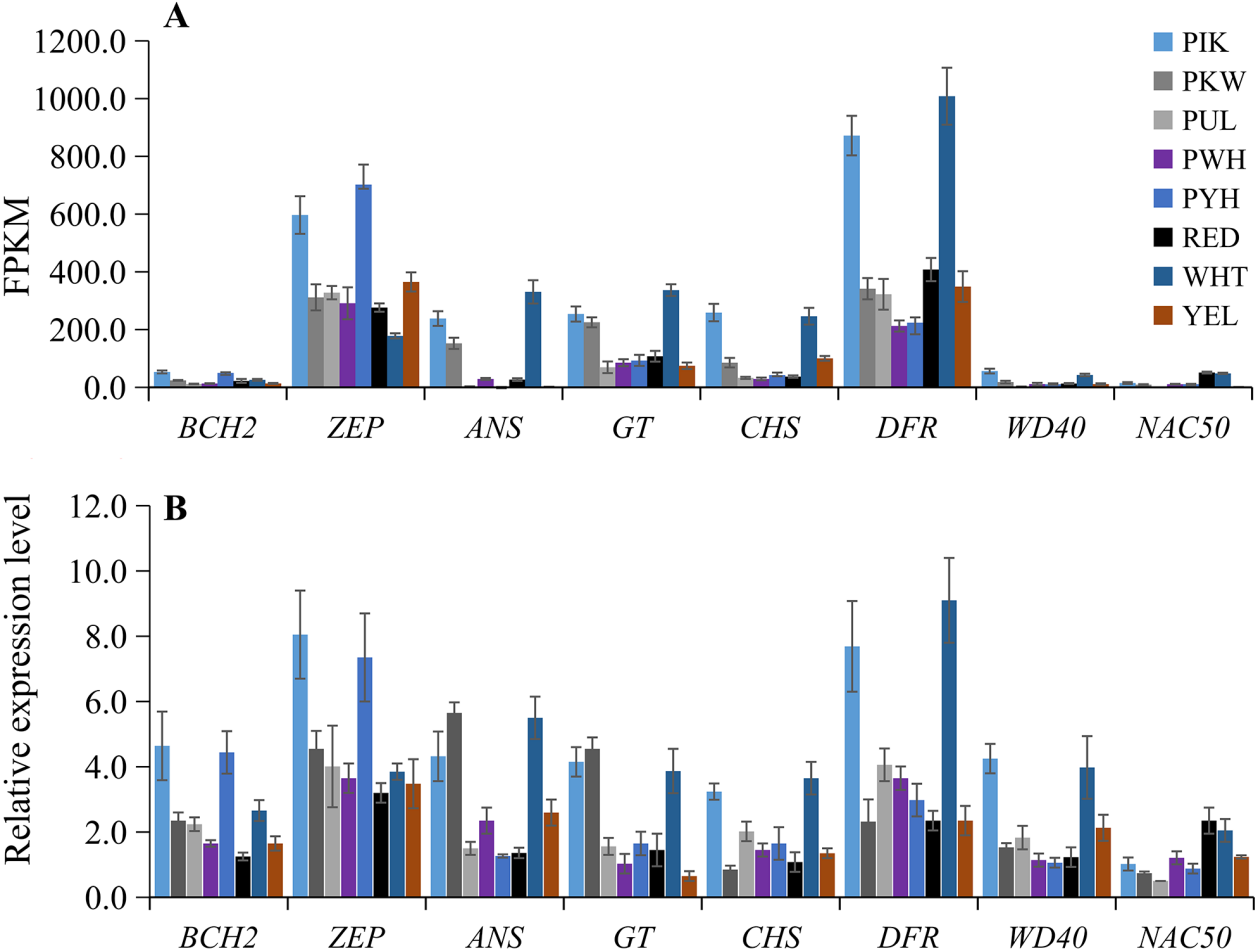
Expression patterns of eight selected unigenes in eight colors of *L. polyphyllus*. (A) Expression level of eight unigenes are based on the fragments per kilobase of transcripts per million mapped fragments (FPKM) value. (B) Relative expression level of eight unigenes obtained by quantitative real-time PCR (qRT-PCR) analysis. The error bars represent the SD from three replicates.

To verify the accuracy of the transcriptome sequencing data, the expression levels of eight candidate unigenes related to carotenoid and anthocyanin biosynthesis were checked using qRT-PCR with three biological replicates. The primers are listed in Table S8. The qRT-PCR results showed that the up-regulated unigenes included *BCH2* and *ZEP* in PIK and PYH, *WD40* in PIK and WHT, *NAC50* in WHT and RED, *ANS* and *GT* in PIK, PKW and WHT, and *CHS* and *DFR* in PIK and WHT (Fig. 6B; Table S9). These results were consistent with those obtained from the expression profiles determined from the RNA-seq data (Fig. 6A). Therefore, the RNA-seq and qRT-PCR analysis results were of high reliability and could be used for advanced research on the genes involved in carotenoid and anthocyanin accumulation in different colors of *L. polyphyllus*.

## Discussion

*L. polyphyllus* is native to North America and spread to Europe, Australia, New Zealand and Chile (Klinger et al., 2020). It was introduced in China as an ornamental plant, and genetic improvement of its color has been a major goal of current studies. RNA-seq is a routine experimental method to identify candidate genes, quantify gene expression and dissect the molecular basis of horticultural traits. For instance, numerous genes related to carotenoid and anthocyanin biosynthesis have been identified by RNA-seq in *Hibiscus cannabinus* (Lyu et al., 2020), *Camellia sinensis* (Rothenberg et al., 2019), *Michelia maudiae* (Lang et al., 2019), *Camellia nitidissima* (Zhou et al., 2017), and *Vitis davidii* (Sun et al., 2016). Although the transcriptome data of *Lupinus luteus* have been reported (Parra-González et al., 2012; Glazinska et al., 2017), very limited RNA-seq data are available for *L. polyphyllus* and other lupine species.

In this study, a transcriptome analysis was performed on eight colors of *L. polyphyllus*. A total of 175,392 transcripts and 89,124 unigenes were assembled, and the annotation information of 54,823 unigenes was obtained from six public databases. The number of assembled transcripts and annotated unigenes was larger than that obtained by RNA sequencing in *Lupinus luteus* (Parra-González et al., 2012). One possible explanation is the great variations in gene expression among different lupine species, and the other reason is that improvement in sequencing technology and data availability allows the assembly of more genes and availability of more functional annotation information. The average length of the transcripts and unigenes was 998 bp and 868 bp, respectively, and 80.16% of the unigenes exhibited higher homology to the sequences from *L. angustifolius.* In conclusion, the transcriptome sequencing data of *L. polyphyllus* are reliable, and the generated data can constitute widely useful transcript libraries for further studies of lupine. Anthocyanins and carotenoids play important roles in color formation, and a series of enzymes are involved in their biosynthetic pathways (Grotewold, 2006; Wang et al., 2018). In this study, a total of 177 unigenes related to carotenoid and anthocyanin biosynthesis were identified in the transcriptome. Among them, seven anthocyanin-related unigenes (*ANS*, *ANR*, *GT*, *CHS*, *CHS1A*, and two *DFRs*) and four carotenoid-related unigenes (*BCH1*, *BCH2*, *ZEP,* and *NCED1*) were specifically up- or down-regulated for one or more colors. Interestingly, except for NCED1 and F3′H, other nine unigenes were up-regulated in PIK, suggesting that the coloration of PIK in *L. polyphyllus* may be regulated by multiple major genes. However, the relationship between the expression levels of three carotenoid-related unigenes and PIK remains to be further elucidated, since there has been no report about the presence of carotenoids in PIK flower of *L. polyphyllus*. Three carotenoid-related unigenes (BCH1, BCH2, and ZEP) and two anthocyanin-related unigenes (ANS and GT) were specifically up-regulated in PYH, suggesting that the coloration of PYH in *L. polyphyllus* may be a result of the combined effect of carotenoids and anthocyanins. Interestingly, the key enzyme gene *DFR* in anthocyanin biosynthesis pathway is specifically up-regulated in WHT of *L. polyphyllus*. In general, white flower has low anthocyanin levels, and one possible explanation is that the highest level of DFR expression is false positive. In addition, a total of 505 TFs belonging to the *MYB*, *R2R3-MYB*, *NAC*, *bHLH,* and *WD40* families were identified. In plants, the *MYB-bHLH-WD40* complex is critical for the activation of the anthocyanin and carotenoid biosynthetic pathways (Ramsay & Glover, 2005; Qi et al., 2011). In this study, the TFs of WD40 and MYB113 were up-regulated in PIK; those of NAC2 and MYB3 were up-regulated in WHT; and those of MYB113 were up-regulated in PYH. However, the co-expression patterns with structural genes and functions remain to be further studied. Variations in the expression abundance of these regulatory genes usually lead to color variations in Orchids (Li et al., 2020a; Li et al., 2020b), *Camellia reticulata* (Yao et al., 2016), and *Brassica napus* (Jia et al., 2021). In conclusion, these results suggest that the expression of these significant DEGs plays an important role in the color formation of *L. polyphyllus*. We for the first time report the transcriptome data of *L. polyphyllus* with different colors, providing a rich molecular resource for further research on coloration mechanism. A large number of transcriptomic sequences may provide sufficient data for the screening of key genes in flavonoid, anthocyanin and carotenoid biosynthetic pathways. Future studies may be focused on the expression of important TFs in flowers at different development stages, the correlation between anthocyanin and carotenoid content, and the expression level of candidate genes to further reveal the specific coloration mechanism.

## Conclusions

A transcriptome analysis of eight different colors of *L. polyphyllus* was performed using RNA-Seq technology. A total of 89,124 unigenes were assembled from 1.13 billion high-quality reads, and the functional annotation information of 54,823 unigenes was obtained from six public databases. A total of 76 and 101 unigenes respectively involved in carotenoid and anthocyanin biosynthesis pathways were revealed. A total of 505 TFs regulating carotenoid and/or anthocyanin biosynthesis were obtained. Among them, 17 candidate unigenes were specifically up-regulated for one or more colors of *L. polyphyllus*. Our research results enrich the transcriptome of lupine, and provide a rich molecular resource for research on the coloration mechanism of *L. polyphyllus*.

##  Supplemental Information

10.7717/peerj.13836/supp-1 Supplemental Information 1 Assembled unigenes were functionally classificated by Gene Ontology categorization The unigenes corresponded to three main categories: biological process, cellular component, and molecular function.

10.7717/peerj.13836/supp-2 Supplemental Information 2 Transcriptome sequencing The summary of sequencing information of 24 cDNA libraries of *Lupinus polyphyllus.*

10.7717/peerj.13836/supp-3 Supplemental Information 3 Statistical analysis of unigenes The information of 89,124 assembled unigenes and 54,823 annotated unigenes.

10.7717/peerj.13836/supp-4 Supplemental Information 4 KEGG analysis The KEGG classification statistical table of annotated unigenes.

10.7717/peerj.13836/supp-5 Supplemental Information 5 Differentially expressed genes (DEGs) The expression and functional annotation information of 6700 significant DEGs.

10.7717/peerj.13836/supp-6 Supplemental Information 6 Comparison analysis The differentially expressed genes (DEGs) statistics analysis of 28 comparison groups

10.7717/peerj.13836/supp-7 Supplemental Information 7 Functional annotation The functional annotation information of 101 anthocyanin-related genes and 76 carotenoid-related genes.

10.7717/peerj.13836/supp-8 Supplemental Information 8 The expression level analysis The FPKM value in 8 colors and functional annotation information of unigenes that showed expressional differences in one or more comparison groups

10.7717/peerj.13836/supp-9 Supplemental Information 9 Primers information The primers of candidate genes for qRT-PCR analysis

10.7717/peerj.13836/supp-10 Supplemental Information 10 The qRT-PCR analysis The raw data of relative expression level of eight candicate genes for three biological replicates (nested with three technical replicates)

## Additional Information and Declarations

### DNA Deposition

The following information was supplied regarding the deposition of DNA sequences: Sequence information is available at NCBI: PRJNA783465.

### Data Availability

The following information was supplied regarding data availability: The raw data are available in the Supplementary Files.
