# Supplementary figures and images for: Comparative transcriptome analysis of Lupinus polyphyllus Lindl. provides a rich molecular resource for research on coloration mechanism
Source: PeerJ. 2022 Aug 2;10:e13836. doi: 10.7717/peerj.13836 (PMC9354738; doi:10.7717/peerj.13836)

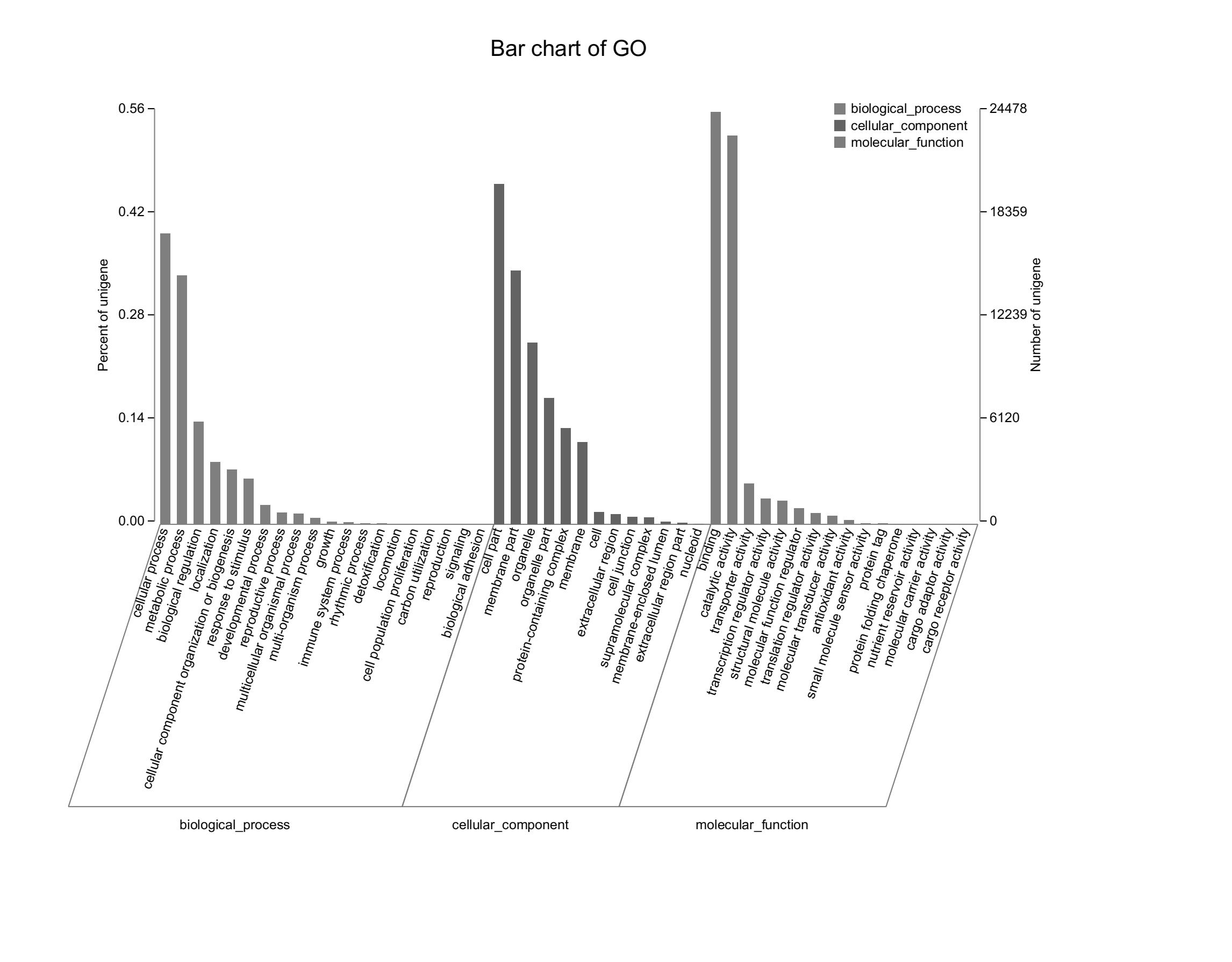

Supplement: Supplemental Information 1 — The unigenes corresponded to three main categories: biological process, cellular component, and molecular function. [file peerj-10-13836-s001.jpg]
