# Supplementary material for: Comparative transcriptome analysis of Lupinus polyphyllus Lindl. provides a rich molecular resource for research on coloration mechanism
Source: PeerJ. 2022 Aug 2;10:e13836. doi: 10.7717/peerj.13836 (PMC9354738; doi:10.7717/peerj.13836)
Supplement: Supplemental Information 2 — The summary of sequencing information of 24 cDNA libraries of Lupinus polyphyllus. [file peerj-10-13836-s002.docx]

**Table S1** **Transcriptome sequencing.** The summary of sequencing information of 24 cDNA libraries of *Lupinus polyphyllus*

| Sample | Raw reads | Clean reads | Error rate (%) | Q30(%) | GC content(%) |
| --- | --- | --- | --- | --- | --- |
| PIK1 | 43193888 | 42462242 | 0.026 | 93.11 | 42.62 |
| PIK2 | 45685834 | 44891638 | 0.0247 | 94.43 | 42.67 |
| PIK3 | 44097908 | 43335956 | 0.0245 | 94.58 | 42.87 |
| PKW1 | 50006218 | 49561014 | 0.0245 | 94.58 | 42.84 |
| PKW2 | 45045444 | 44296154 | 0.0243 | 94.79 | 42.7 |
| PKW3 | 47700448 | 46878854 | 0.0244 | 94.73 | 42.65 |
| PUL1 | 47795610 | 46940028 | 0.0247 | 94.43 | 43.12 |
| PUL2 | 55720958 | 55152928 | 0.0248 | 94.31 | 42.82 |
| PUL3 | 50777514 | 49893494 | 0.0245 | 94.65 | 43.19 |
| PWH1 | 47745244 | 46934504 | 0.0246 | 94.54 | 42.87 |
| PWH2 | 49516496 | 48661202 | 0.0247 | 94.43 | 42.92 |
| PWH3 | 46599208 | 45759774 | 0.0247 | 94.43 | 42.87 |
| PYH1 | 46642634 | 45839204 | 0.0248 | 94.28 | 42.35 |
| PYH2 | 51543544 | 50643892 | 0.0247 | 94.37 | 42.52 |
| PYH3 | 45357798 | 44595930 | 0.0245 | 94.6 | 42.71 |
| RED1 | 48120708 | 47245226 | 0.0248 | 94.3 | 42.83 |
| RED2 | 49352090 | 48515368 | 0.0245 | 94.65 | 43.09 |
| RED3 | 46736220 | 45890826 | 0.0245 | 94.61 | 43.01 |
| WHT1 | 49042460 | 48170230 | 0.0248 | 94.31 | 42.99 |
| WHT2 | 44699218 | 43894028 | 0.0245 | 94.64 | 42.91 |
| WHT3 | 48361084 | 47537622 | 0.0244 | 94.67 | 42.73 |
| YEL1 | 52121014 | 51152384 | 0.0248 | 94.33 | 42.9 |
| YEL2 | 46849814 | 46000552 | 0.0247 | 94.38 | 42.57 |
| YEL3 | 49498362 | 48624520 | 0.0244 | 94.75 | 42.98 |
| Total/Average | 1152209716 | 1132877570 | 0.0246 | 94.45 | 42.82 |
