# Supplementary material for: Comparative transcriptome analysis of Lupinus polyphyllus Lindl. provides a rich molecular resource for research on coloration mechanism
Source: PeerJ. 2022 Aug 2;10:e13836. doi: 10.7717/peerj.13836 (PMC9354738; doi:10.7717/peerj.13836)
Supplement: Supplemental Information 9 — The primers of candidate genes for qRT-PCR analysis [file peerj-10-13836-s009.doc]

Supplementary Table 8 **Primers information.**The primers of candidate genes for qRT-PCR analysis

| Gene | Forward and Reverse primer sequences | Length | Annealing (℃) |
| --- | --- | --- | --- |
| *NAC50* | F:AATCCTTCCGTTTTGACCCCATCG | 24 | 57.2 |
|  | R:TCCAAATCCCTCGTCTTCAGTTTCG | 25 | 56.8 |
| *WD40* | F:ATGCTTTGGTTCGGTCTCGTTCC | 23 | 56.0 |
|  | R:AAGTTCTCGTAAGTCGCTGCTTCG | 24 | 53.0 |
| *DFR* | F:GCCAAGGATCTAGCTGAGAACAACC | 25 | 57.0 |
|  | R:AATCATAGCAGCAGCACCATCACC | 24 | 56.5 |
| *CHS* | F:GCCAAGGATCTAGCTGAGAACAACC | 25 | 55.0 |
|  | R:AATCATAGCAGCAGCACCATCACC | 24 | 54.5 |
| *GT* | F:GGCACCACCTCCACATGAGATTG | 23 | 61.2 |
|  | R:TGAAGAAACCCACTTGGCAAGAGAC | 25 | 57.5 |
| *ANS* | F:TCACAACATGGTTCCAGGTCTTCAG | 25 | 60.4 |
|  | R:TGCTCAAGATTTCAAGGGTGTCACC | 25 | 59.8 |
| *ZEP* | F:TATTGGGCTAGATGGGCTCATAGGG | 25 | 59.5 |
|  | R:AGAGGAGAGCAATAGCAGGGACAG | 24 | 58.5 |
| *BCH2* | F:TATTGGGCTAGATGGGCTCATAGGG | 25 | 60.2 |
|  | R:AGAGGAGAGCAATAGCAGGGACAG | 24 | 61.2 |
| *LpActin* | F:ATGGCATGAGGGAGGGCATACC | 22 | 58.6 |
|  | R:GCTAGTGGTCGTACAACTGGGATTG | 25 | 58.5 |
